# Adaptation and Validation of the Motivated Strategies for Learning Questionnaire for Spanish Adolescents

**DOI:** 10.3390/ejihpe11010012

**Published:** 2021-02-13

**Authors:** Adrián Segura-Robles, Antonio-José Moreno-Guerrero, María-Elena Parra-González, Jesús López-Belmonte

**Affiliations:** 1 Department of Research Methods and Diagnosis in Education, University of Granada, 51001 Ceuta, Spain; adrianseg@ugr.es (A.S.-R.); elenaparra@ugr.es (M.-E.P.-G.); 2 Department of Didactics and School Organization, University of Granada, 51001 Ceuta, Spain; ajmoreno@ugr.es

**Keywords:** validation, questionnaire, motivation in learning, intrinsic motivation, extrinsic motivation

## Abstract

This work adapts and validates the scale of the motivated strategies for learning questionnaire (MSLQ), which is used to measure motivation. For this, an instrumental design was carried out with the purpose of analyzing the psychometric properties of the instrument. The sample consisted of 307 participants enrolled in compulsory secondary education. Reliability with fit indices were good in model B (proposed) with composite reliability, global reliability index, and Cronbach’s alpha. The original model (A) presented small problems that had to be adjusted when carrying out the translation. We concluded that adaptation and subsequent validation of the MSLQ instrument into a Spanish context was positive. In this sense, adequate adjustment rates have been achieved. However, in its contextual adequacy, the need arises to modify the presentation of the items alluding to intrinsic motivation due to the difficulty of measuring such a construct. Among the implications reached in this study is the possibility of having a validated instrument for the Spanish adolescent context to measure motivation on educational aspects. Furthermore, this tool can serve as the basis for the design of other instruments that measure this construct in other age ranges.

## 1. Introduction

Student learning is the purpose of any pedagogical act [1]. This fact appears to be simple yet nonetheless carries a set of implications that need to be properly developed [2]. One of these aspects is motivation [3,4].

There is consensus in the scientific literature when it comes to defining motivation [5]. This is considered as an individual state or condition of each person that promotes a series of actions in the subject [6]. It can be positive, prompting individuals to approach stimuli, or negative, generating an opposite attitude, i.e., moving away from the stimulus that causes it [7]. Currently, there are two kinds of motivation: intrinsic motivation (IM) and extrinsic motivation (EM) [8]. The actions that an individual performs to satisfy his own needs are considered intrinsic motivation [9], i.e., why a person performs certain actions due to his own interests [10]. On the other hand, extrinsic motivation moves an individual to carry out actions in order to satisfy his environment [11]. In this case, the person tries to gain recognition of his environment, be it near or distant [12].

Several studies in the educational field have analyzed intrinsic motivation [13,14], all of which focused on situations of choice and novelty of suggested content [15]. Moreover, they looked at carrying out pedagogical actions with techno-pedagogical resources [16] or reading development via students choosing texts [17]. It has been shown that intrinsic motivation affects men and women differently, especially when there is a public assessment of academic performance [18].

The studies on extrinsic motivation apply to educational settings [19]. Many of these studies focus on the effect of mobbing [20] on the development of learning through services [21], the influence of physical exercise [22], collaboration with sick people [23], or learning other languages [24]. Again, as with analogous motivation, gender also influences extrinsic motivation, especially in connection to stressful situations [25]. 

Focusing on motivation in the educational field, it can be considered as an essential part of self-regulation, and thus a fundamental aspect for learning acquisition [26]. This self-regulation can come from metacognitive motivating and behavioral actions generated by students engaged directly with their own learning [27]. Thanks to the self-regulation of learning, students gain an understanding of their own will [28] through their involvement in pedagogical acts, with no need to generate inner conflicts [29]. It can be said that self-regulation triggers both academic and learning skills [30]. Several studies have, as such, investigated motivation via different approaches, such as the motivation of achievements [31], the motivation of students toward learning [32], self-regulated learning, and work motivation [33].

In the scientific literature, there are several questionnaires or scales to measure student motivation, among which is the motivated strategies for learning questionnaire (MSLQ) [34]. This instrument analyzes both intrinsic and extrinsic motivation [35]. In this tool, intrinsic motivation refers to the internal approval of the students themselves [36]. In contrast, extrinsic motivation refers to the acquisition of academic achievements reflected through qualifications or external approvals in their environment [20]. It must be kept in mind that both motivations must be considered as opposite poles of a unidimensional continuum. This continuum goes from the exterior to internal self-regulation, i.e., it ranges from extrinsic motivation to intrinsic motivation [23].

The MSLQ focuses primarily on the motivational orientation and learning strategies of students in secondary and higher education [37]. This instrument has been translated and adapted in several languages and can be used to identify students’ motivation in higher education [38]. This instrument is divided into two subscales [37]. One of them analyzes intrinsic motivation, oriented towards learning and focusing on the internal approval of the subject. The other analyzes extrinsic motivation, related to grades, and focusing on the subject’s external approval [39]. The analysis of both dimensions can be carried out separately, as they do not necessarily complement each other [38]. This instrument is intended to be used in several higher education courses, not only in one [40]. Furthermore, this instrument has been analyzed from a statistical point of view to find out its psychometric properties. In all of them, the reliability of the instrument has been found to be adequate, with values close to 0.70 in both dimensions. It should be noted that these results are the result of the analysis carried out in the validations and reliability of several instruments via a meta-analysis [38]. This instrument has also been used to analyze various aspects and has been used in various contexts [41,42,43,44], which makes it in itself a widely and internationally used instrument [45]. 

In other studies, the MSLQ instrument has been analyzed on the basis of self-determination theory [46], where the two dimensions of the MSLQ scale have been considered as two opposite poles (both extrinsic and intrinsic-dry motivation) on a unidimensional continuum of motivation [47], indicating that student motivation evolves from extrinsic motivation to intrinsic motivation [48]. This comparison may be similar to femininity and masculinity, where the person either exhibits one type of majority motivation or the other, but not both at the same time [49]. 

The Nielsen questionnaire has been useful to determine how both types of motivation affect students in various reviews, according to gender [50], age [51], nationality [52], and culture [53]. Moreover, it has been used to set motivational stereotypes [54]. Most of these reviews have used quasi-experimental methods by applying a pretest–posttest.

Based on all of the above, this work aimed to continue the path started by previous studies that approached intrinsic and extrinsic motivations in different contexts. Therefore, this research attempted to adapt Nielsen’s MSLQ into a Spanish-speaking context. This task was carried out with the purpose of designing and introducing a valid and reliable instrument into the scientific community, and to obtain results related to motivation in Spanish students.

## 2. Materials and Methods

### 2.1. Design

For carrying out this research, an instrumental design was used. This type of design has been used for researches especially when the purpose is to analyze the psychometric properties of the instruments. In this case, the main goal was to perform the validation of an instrument [55].

### 2.2. Instrument

The instrument used was the MSLQ IM and EM subscales or motivational strategies scale. This scale was developed and validated in the English language by Nielsen [34]. The answer options are Likert type (1 = not at all, 2 = to a small degree, 3 = to some degree, 4 = to a large degree, 5 = perfectly). It consists of two main dimensions around the experience of the participants in online activities or work environments. The first dimension is called intrinsic motivation (IM), i.e., studying for the purpose of learning or internal approval, while the second dimension is called extrinsic motivation (EM), i.e., studying for grades or external approval.

### 2.3. Instrument Translation

There are no clear methodological guidelines on how to translate the questionnaires [56], especially when they need cultural adaptations [57]. These adaptations, for example, are used when cultural contexts (e.g., key distance, mental structures) may be different and the literal translation of an item loses its meaning. As for this study, the guidelines set by [58] regarding translation and adaptation of scales were followed. The steps followed to translate this instrument are specified within the next phases below:

- Phase 1. First, two bilingual experts reviewed the structure of the document in English and analyzed whether it was feasible to translate the questionnaire into Spanish.
- Phase 2. Then, the authors, who had a high proficiency level in English and were native Spanish speakers, proceeded to translate the questionnaire into Spanish.
- Phase 3. Afterward, the experts and two monolingual researchers reviewed the translation and proposed various modifications.
- Phase 4. Finally, the bilingual experts translated the scale into Spanish and subsequently checked its concordance and coherence with the translations that have been made and those made by the same authors; thus, in this way, a scale as faithful as possible to the original was obtained.

### 2.4. Procedure

The data collection process began in March 2020. The administration of the questionnaire was carried out using Google Forms. This allowed researchers to access participants more quickly and efficiently. Once all the data were collected, they were downloaded in the form of a matrix. The next step was to input the data into the SPSS statistical analysis software, version 25, for further analysis. Within this research, all principles and criteria were established using the Code of Good Research Practices, as established by the Declaration of Helsinki. Similarly, all participants were aware of the study objectives, informed consent was obtained, and their anonymity was preserved. Likewise, a regional Ethics Committee made up of researchers external to the research approved the study.

### 2.5. Participants

The sample consisted of 307 students, who took part in the research during the 2019–2020 academic year. The average age of the participants was 14 years old, and they belonged to the compulsory secondary education stage in Spain. The school was located in the autonomous city of Ceuta, a Spanish border city. Ceuta is located in the north of the African continent but politically belongs to the Government of Spain. Ceuta stands out for the coexistence of four cultures (Christian, Muslim, Hebrew, and Hindu). The sampling technique was intentional [59]. Two large educational centers of the aforementioned city participated. These centers present two totally different natures. One is of a public nature and the other is arranged by the Ministry of Education and Vocational Training. The basic descriptors of the sample are found in Table 1, where it can be observed that, in each group, there were at least five subjects per item, which was an essential requirement for the validation of the instrument [60,61].

## 3. Results

### 3.1. Exploratory Study

Before carrying out a confirmatory factor analysis (CFA), the questionnaire’s factor structure was tested via the exploratory factor analysis (EFA) to confirm its structure. To carry out this analysis, we verified the sufficiency of the sample through the KMO test (Kaiser–Meyer–Olkin measure of sampling adequacy). The results obtained (KMO (28) = 0.730; *p* = 0.000) confirmed the possibility of performing the said analysis [62,63].

Table 2 shows the matrix of rotated components after performing EFA with Varimax rotation, standard in social science studies. This kind of analysis is routinely used for questionnaire validation, particularly in psychology and the social sciences [64]. The results show that two factors appear after the analysis (factors 1 and 2), as in the original model and that the items are grouped almost exactly with the originals. Load factors showed values above 0.40, demonstrating their suitability. Values with an item weight of <0.40 were eliminated as they were not statistically relevant. Only item IM3, which referred to content quality effects on motivation, did not seem to fit the original dimension in which it was proposed. A possible explication is that “intrinsic motivation should be limited to referring to the pleasure gained from an activity, divorced from any further elements” [65] (p. 1). 

**Figure 1:**
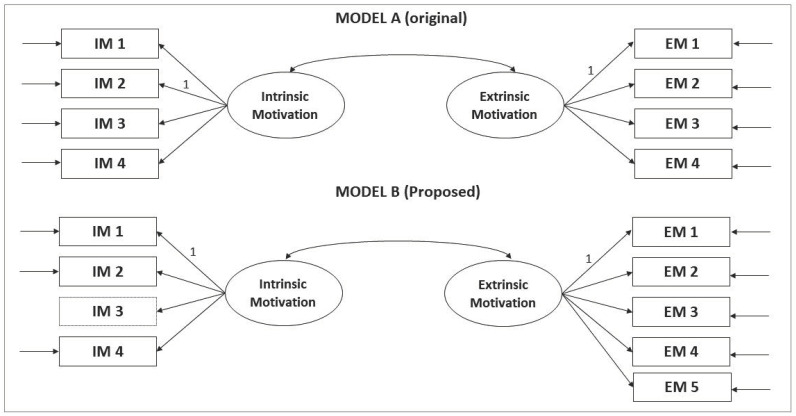
Factorial tested models, model A (original) and model B (proposed).

After analyzing the item’s wording, a factorial structure sample was established, which will be tested in future analyses. Once the original structure of the scale was tested, two different models were settled. On the one hand, model A maintained the original structure and dimensionality. On the other, model B had item IM3 become EM5 (Figure 1). 

### 3.2. Confirmatory Factor Analysis

To assess the fit of the model, a chi-square value was used for both models (i.e., model A—χ^2^ = 66.902; gl = 0.19; *p* = 0.000, model B—χ^2^ = 35.474; gl = 0.19; *p* = 0.012). This value was very sensitive to the sample size [66], and thus it had to be completed with classic indicators for goodness of fit. In this instance, we used the RMSEA (root-mean-square error of approximation), the comparative indices NFI (normed fit index), GFI (goodness of fit index), TLI (Tucker–Lewis index), CFI (comparative fit index), and IFI (incremental fit index). These indicators allowed us to know how a proposed model adjusts to its original construction. The results are shown together with the values proposed by the literature to facilitate their understanding (Table 3).

Regarding reliability, the classical Cronbach alpha index was used. Although the composite reliability indicator (CF) was added due to the statistical weakness of the alpha indicator, acceptable values being those that exceed 0.7 [67]. Another of the most commonly used indicators to evaluate scales in social sciences is discriminant validity [68]. Although the extracted mean-variance (EMV) has usually been used as a reference indicator for discriminant validity, it showed little robustness in the current research [69]. Therefore, the use of the HTMT indicator (heterotrait–monotrait ratio of correlations) was used, as proposed by [70].

As can be seen in Table 4, model A (original) presented several problems regarding its reliability indicators. Thus, we found problems in the extrinsic dimension with CR = 0.631 and for alpha results in intrinsic motivation with a value of 0.612. On the other hand, model B (proposed) obtained satisfactory values for most used indicators (CR < 0.70; MSV = 0.111; HTMT = 0.414). As with the original model, Cronbach’s alpha for the intrinsic motivation dimension was below the levels recommended in the scientific literature, alpha < 0.7. All these results confirm that the previously proposed model B has good reliability, even better than the original model, which is why this second structure is proposed as a final questionnaire for use in the Spanish or Latin American context.

Finally, Table 5 shows the correlations between every factor proposed in the original model. As can be seen, intrinsic and extrinsic motivation were almost related in a positive way (r = 0.94, *p* < 0.05). Showing that although they seem to be the same thing, they are two different constructs that must be analyzed separately.

## 4. Discussion and Conclusions

As the scientific literature shows, the concept of motivation assumes a relevant value in general education [3], as well as the processes of teaching and learning, in particular [26]. This was summarized in the importance of this construct for students to achieve high levels both in their academic performance and in their attitude toward their studies [30,31,32,33]. There have been many studies collected in the scientific literature that analyze the motivation construct [71,72,73,74,75,76]. This leads to generating a relevant tool that can be adapted to each of the training contexts in Spain, i.e., to various subjects and contents in which to assess the motivation of students after the application of a certain teaching and learning methodology.

To understand the daily motivation of students, it is essential to use validated instruments, i.e., tools that allow for the collection of precise data on the question of art [77]. Another particular of great importance is the instrument’s adjustment to context, since cultural and geographical differences may determine the accuracy and precision of the obtained results [78]. Consequently, the adaptation of tools designed by other researchers for other contexts becomes an interesting and exploratory field of study. All this has the purpose to offer the scientific community a wide spectrum of instruments that serve to measure specific particular aspects and can be adapted to different contexts. This will allow the narrowing of research biases, as well as the attainment of precise studies with reliable and relevant results [79].

The results achieved in this adaptation and subsequent validation are suitable for applying the MSLQ instrument in a Spanish context. Acceptable but not excellent fit indices were obtained, especially for the original model. This was in line with other adaptations and validations that the original instrument made to various contexts and specific areas of knowledge [80,81,82,83,84,85]. In such publications on the different validations carried out with the MSLQ, different incidents and burdens on certain items were reflected that condition the reliability values. This made us reflect on the importance of validating the instrument for each geographic region and each pedagogical subject. This is a consequence of the peculiarities of each study population.

Although the original instrument proposed by Nielsen presented relevant psychometric properties, an analysis made for a Spanish context was needed to modify the wording of the items concerning intrinsic motivation. This came from the difficulty of measuring this specific construct exhibited in previous studies [86]. This difficulty was exponentially accented in ages such as adolescence [87], which was the composition of the sample analyzed in this research. In the same way, the literature displays the weakness of accurately measuring intrinsic motivation [88,89]. All of this indicates that the results should be interpreted cautiously. Our findings show the relationship between the two analyzed types of motivation [90].

Therefore, we concluded that the adaptation and validation of the MSLQ to a Spanish context (Appendix A) obtained psychometric values and properties that positioned it as a valid and reliable instrument to collect information on the motivation of Spanish adolescent students.

This research had several limitations. First: access to the sample. Once an educational center with an adequate volume of participants needed to validate the tool was found, the COVID-19 pandemic occurred. This delayed the study and caused student participation to suffer. Many students did not have the necessary means at home to complete the questionnaire. To solve this problem, the school provided electronic resources to the most disadvantaged students. Therefore, this research was conditioned by the situation generated by the pandemic.

As a future line of research, one should apply the designed instrument in different regions of the Spanish territory. This has the purpose of distinguishing the motivation of students during a pandemic. Therefore, this tool attained a fundamental value in current education, conditioned and limited in many aspects, by the adaptation of the training processes derived from the health crisis. In short, the knowledge of the motivation of today’s students rises as a relevant factor due to the methodological changes that occurred. All of this demands tools adapted to different contexts, such as the one offered here.

## 5. Theoretical and Practical Implications

This study developed a set of implications of both a theoretical and practical nature. Regarding the theoretical implications, the questionnaire designed and adapted to the Spanish context supposed the appearance of a validated instrument for the preparation of studies on the motivation of Spanish adolescents in educational aspects, which was relevant for the educational community. Motivation, as it has been verified in the presented literature, is a construct that has been studied in recent years as a consequence of the appearance of new training approaches, as well as of new spaces generated as a consequence of the COVID-19 pandemic. Therefore, a recent validated and reliable tool to measure motivation in Spanish students is positioned as one more element to reduce this gap in the Spanish scientific literature.

With regards to the practical implications of this work, the availability of a validated and appropriate tool for a certain context allows educational institutions to identify the motivation of students in aspects concerning various actions or training programs. This is the order of the day due to the use of active methodologies (e.g., gamification, escape rooms, flipped learning, ubiquitous learning) and emerging technologies (e.g., robotics, augmented reality, virtual reality) penetrating learning spaces. This allows for the opportunity to transform traditional teaching and learning processes, which must be valued at a motivational level.

This instrument being short in length allows it to be used as a complement to other instruments. This will allow teachers or the scientific community to carry out data collection covering various dimensions.

Also, this instrument is valid for educational contexts where there are Latin American students both in Europe and in Latin America. In addition, this tool can be used in those countries where Spanish for foreigners or Spanish as a second language is taught.

By taking into consideration this instrument and the results that can be obtained with its use, professionals can use the information to improve motivation levels and work on them. Likewise, this tool can serve as support for the design and development of other instruments that are made to measure motivation in other educational stages, many of which cover additional age ranges. This is important as motivation can ease student learning, which is needed at all educational stages. 

## Figures and Tables

**Table 1:** Characteristics of the participating sample.

		n	%
**Gender**	Female	173	56.4%
	Male	134	43.6%
**Course**	First level	65	21.2%
	Second level	73	23.8%
	Third level	107	34.9%
	Fourth level	62	20.2%

**Table 2:** Rotated factorial structure and original factorial load.

	Factors	
	1	2
**Intrinsic**		
MotInt1		0.841
MotInt2		0.844
MotInt3	0.510	
MotInt4		0.556
**Extrinsic**		
MotExt1	0.781	
MotExt2	0.754	
MotExt3	0.615	
MotExt4	0.684	

Note: Columns 1 and 2 indicate the number of factors obtained and the right column indicates the original factors.

**Table 3:** The goodness of fit indices for the proposed model and cuts marked by the literature. RMSEA (root-mean-square error of approximation), the comparative indices NFI (normed fit index), GFI (goodness of fit index), TLI (Tucker–Lewis index), CFI (comparative fit index), and IFI (incremental fit index).

	CMIN/DF	NFI	NNFI/TLI	CFI	IFI	RMSEA
**Model A**	3521	0.860	0.843	0.893	0.895	0.091
**Model B**	1867	0.926	0.946	0.663	0.964	0.053
**Literature**	Between 1–3	≥0.90	≥0.90	≥0.90	≥0.90	≤0.08

**Table 4:** Reliability and validity measures based on the analyzed models.

	Alpha (α)	CR	MSV	HTMT
**Model A**	0.701			
**Intrinsic**	0.612	0.710	0.125	0.521
**Extrinsic**	0.700	0.631	0.125	
**Model B**	0.701			
**Intrinsic**	0.661	0.718	0.111	0.414
**Extrinsic**	0.704	0.700	0.111	

**Table 5:** Correlation between intrinsic and extrinsic motivation in the proposed model.

	Extrinsic	*p*
**Intrinsic**	0.33	0.010

## Data Availability

Data is contained within the article.

## Appendix A

**Table A1:** Spanish version of the translated questionnaire.

	Escala				
Dimensiones	1	2	3	4	5
**Motivación Intrínseca**					
Prefiero material de clase que realmente sea un reto para poder aprender cosas nuevas					
Prefiero material de clase que despierte mi curiosidad, aunque sea difícil de aprender					
Lo más satisfactorio para mí es entender los contenidos de la mejor forma posible					
Cuando tengo oportunidad de elegir, elijo hacer tareas en las que puedo aprender aunque no garanticen una buena nota					
**Motivación Extrínseca**					
Obtener una buena nota en clase es lo más satisfactorio para mí en este momento					
Lo más importante para mí en este momento es mejorar la media de mis notas, así que mi principal preocupación es conseguir una buena nota					
Si puedo, quiero obtener mejores notas que la mayoría de los otros estudiantes					
Quiero hacerlo bien en clase porque es importante mostrar mi habilidad a mi entorno (familia, amigos, profesores u otras personas)					
